# Supplementary material for: Time-series transcriptome analysis identified differentially expressed genes in broiler chicken infected with mixed Eimeria species
Source: Front Genet. 2022 Aug 8;13:886781. doi: 10.3389/fgene.2022.886781 (PMC9393255; doi:10.3389/fgene.2022.886781)
Supplement: Supplementary file 2 [file DataSheet1.ZIP › 7dpi_KEGG.Gsea.1625115961950/KEGG_HUNTINGTONS_DISEASE.html]

Details for gene set KEGG\_HUNTINGTONS\_DISEASE[GSEA]

|  || Dataset | TMM\_7dpi\_gct\_format\_7dpi\_gct\_format.Class\_4dpi.cls #PC\_versus\_NC.Class\_4dpi.cls #PC\_versus\_NC\_repos |
| Phenotype | Class\_4dpi.cls#PC\_versus\_NC\_repos |
| Upregulated in class | 1 |
| GeneSet | KEGG\_HUNTINGTONS\_DISEASE |
| Enrichment Score (ES) | 0.5114907 |
| Normalized Enrichment Score (NES) | 2.6486828 |
| Nominal p-value | 0.0 |
| FDR q-value | 0.0 |
| FWER p-Value | 0.0 |
Table: GSEA Results Summary

  

Fig 1: Enrichment plot: KEGG\_HUNTINGTONS\_DISEASE      
 Profile of the Running ES Score & Positions of GeneSet Members on the Rank Ordered List

  

| SYMBOL | TITLE | RANK IN GENE LIST | RANK METRIC SCORE | RUNNING ES | CORE ENRICHMENT || 1 | PPARG | na | 83 | 1.306 | 0.0168 | Yes |
| 2 | GPX1 | na | 190 | 1.058 | 0.0271 | Yes |
| 3 | SLC25A5 | na | 276 | 0.959 | 0.0374 | Yes |
| 4 | COX5A | na | 300 | 0.942 | 0.0526 | Yes |
| 5 | NDUFA2 | na | 303 | 0.935 | 0.0694 | Yes |
| 6 | NDUFB6 | na | 307 | 0.933 | 0.0862 | Yes |
| 7 | NDUFS5 | na | 308 | 0.932 | 0.1031 | Yes |
| 8 | NDUFB7 | na | 323 | 0.915 | 0.1186 | Yes |
| 9 | PPARGC1A | na | 339 | 0.894 | 0.1336 | Yes |
| 10 | NDUFB2 | na | 348 | 0.885 | 0.1490 | Yes |
| 11 | NDUFAB1 | na | 356 | 0.876 | 0.1644 | Yes |
| 12 | NDUFA7 | na | 371 | 0.857 | 0.1788 | Yes |
| 13 | NDUFC2 | na | 394 | 0.834 | 0.1921 | Yes |
| 14 | UQCR10 | na | 444 | 0.785 | 0.2023 | Yes |
| 15 | CYCS | na | 475 | 0.763 | 0.2136 | Yes |
| 16 | COX6A1 | na | 480 | 0.760 | 0.2271 | Yes |
| 17 | UQCR11 | na | 485 | 0.754 | 0.2405 | Yes |
| 18 | UQCRB | na | 487 | 0.753 | 0.2541 | Yes |
| 19 | COX7A2 | na | 499 | 0.743 | 0.2667 | Yes |
| 20 | CASP8 | na | 514 | 0.732 | 0.2789 | Yes |
| 21 | POLR2F | na | 609 | 0.681 | 0.2833 | Yes |
| 22 | NDUFS3 | na | 623 | 0.675 | 0.2945 | Yes |
| 23 | NDUFB3 | na | 656 | 0.657 | 0.3038 | Yes |
| 24 | NDUFS8 | na | 661 | 0.653 | 0.3153 | Yes |
| 25 | NDUFS6 | na | 686 | 0.642 | 0.3250 | Yes |
| 26 | SDHD | na | 708 | 0.632 | 0.3347 | Yes |
| 27 | NDUFA1 | na | 742 | 0.616 | 0.3431 | Yes |
| 28 | UQCRHL | na | 788 | 0.594 | 0.3502 | Yes |
| 29 | NDUFA5 | na | 794 | 0.593 | 0.3605 | Yes |
| 30 | SDHB | na | 840 | 0.575 | 0.3672 | Yes |
| 31 | NDUFB5 | na | 902 | 0.554 | 0.3722 | Yes |
| 32 | HDAC1 | na | 908 | 0.552 | 0.3818 | Yes |
| 33 | COX6C | na | 930 | 0.546 | 0.3900 | Yes |
| 34 | NDUFV3 | na | 988 | 0.527 | 0.3948 | Yes |
| 35 | NDUFB4 | na | 1015 | 0.517 | 0.4020 | Yes |
| 36 | POLR2L | na | 1038 | 0.508 | 0.4094 | Yes |
| 37 | COX7B | na | 1055 | 0.501 | 0.4171 | Yes |
| 38 | NDUFA6 | na | 1079 | 0.492 | 0.4241 | Yes |
| 39 | NDUFA8 | na | 1080 | 0.492 | 0.4331 | Yes |
| 40 | UQCRQ | na | 1103 | 0.484 | 0.4401 | Yes |
| 41 | NDUFS2 | na | 1124 | 0.477 | 0.4471 | Yes |
| 42 | COX4I1 | na | 1125 | 0.476 | 0.4557 | Yes |
| 43 | SDHC | na | 1135 | 0.473 | 0.4636 | Yes |
| 44 | NDUFB10 | na | 1215 | 0.450 | 0.4651 | Yes |
| 45 | VDAC2 | na | 1244 | 0.443 | 0.4708 | Yes |
| 46 | UQCRFS1 | na | 1263 | 0.437 | 0.4773 | Yes |
| 47 | ATP5PB | na | 1267 | 0.436 | 0.4849 | Yes |
| 48 | NDUFV1 | na | 1307 | 0.428 | 0.4894 | Yes |
| 49 | NDUFB8 | na | 1330 | 0.423 | 0.4953 | Yes |
| 50 | NDUFS7 | na | 1371 | 0.412 | 0.4994 | Yes |
| 51 | NDUFV2 | na | 1395 | 0.408 | 0.5049 | Yes |
| 52 | UQCRC2 | na | 1700 | 0.332 | 0.4854 | Yes |
| 53 | IFT57 | na | 1718 | 0.328 | 0.4899 | Yes |
| 54 | POLR2J | na | 1733 | 0.324 | 0.4946 | Yes |
| 55 | COX7A2L | na | 1761 | 0.316 | 0.4981 | Yes |
| 56 | SOD2 | na | 1821 | 0.302 | 0.4986 | Yes |
| 57 | NDUFB9 | na | 1842 | 0.299 | 0.5024 | Yes |
| 58 | DCTN2 | na | 1860 | 0.296 | 0.5063 | Yes |
| 59 | DNAL4 | na | 1978 | 0.275 | 0.5015 | Yes |
| 60 | NDUFB1 | na | 1995 | 0.272 | 0.5051 | Yes |
| 61 | CASP9 | na | 2022 | 0.268 | 0.5078 | Yes |
| 62 | POLR2E | na | 2072 | 0.258 | 0.5084 | Yes |
| 63 | POLR2I | na | 2091 | 0.255 | 0.5115 | Yes |
| 64 | PLCB4 | na | 2151 | 0.245 | 0.5110 | No |
| 65 | TFAM | na | 2295 | 0.223 | 0.5030 | No |
| 66 | UQCRC1 | na | 2326 | 0.217 | 0.5044 | No |
| 67 | POLR2K | na | 2334 | 0.216 | 0.5078 | No |
| 68 | CLTB | na | 2605 | 0.177 | 0.4883 | No |
| 69 | POLR2D | na | 2681 | 0.167 | 0.4850 | No |
| 70 | NDUFA4 | na | 2726 | 0.160 | 0.4842 | No |
| 71 | POLR2H | na | 2813 | 0.149 | 0.4797 | No |
| 72 | NDUFA9 | na | 2879 | 0.141 | 0.4768 | No |
| 73 | NDUFA10 | na | 3412 | 0.073 | 0.4334 | No |
| 74 | SDHA | na | 3488 | 0.064 | 0.4282 | No |
| 75 | SOD1 | na | 3502 | 0.063 | 0.4283 | No |
| 76 | NDUFS1 | na | 3763 | 0.033 | 0.4070 | No |
| 77 | GNAQ | na | 4006 | 0.004 | 0.3867 | No |
| 78 | PPID | na | 4134 | -0.010 | 0.3762 | No |
| 79 | POLR2C | na | 4136 | -0.010 | 0.3763 | No |
| 80 | NDUFS4 | na | 4402 | -0.040 | 0.3548 | No |
| 81 | TGM2 | na | 4527 | -0.053 | 0.3453 | No |
| 82 | TBPL1 | na | 4594 | -0.059 | 0.3408 | No |
| 83 | TBP | na | 4633 | -0.064 | 0.3388 | No |
| 84 | HIP1 | na | 4672 | -0.067 | 0.3368 | No |
| 85 | HAP1 | na | 4772 | -0.078 | 0.3299 | No |
| 86 | COX7C | na | 4881 | -0.090 | 0.3225 | No |
| 87 | HDAC2 | na | 4971 | -0.100 | 0.3168 | No |
| 88 | DNAL1 | na | 5003 | -0.103 | 0.3161 | No |
| 89 | SP1 | na | 5179 | -0.119 | 0.3035 | No |
| 90 | CREBBP | na | 5240 | -0.125 | 0.3008 | No |
| 91 | SLC25A4 | na | 5331 | -0.133 | 0.2956 | No |
| 92 | VDAC3 | na | 5532 | -0.152 | 0.2816 | No |
| 93 | DCTN1 | na | 5621 | -0.162 | 0.2771 | No |
| 94 | TAF4B | na | 5944 | -0.196 | 0.2536 | No |
| 95 | SLC25A6 | na | 6201 | -0.225 | 0.2361 | No |
| 96 | AP2A2 | na | 6265 | -0.231 | 0.2350 | No |
| 97 | CASP3 | na | 6448 | -0.248 | 0.2243 | No |
| 98 | CREB3L3 | na | 6528 | -0.257 | 0.2223 | No |
| 99 | CREB3L4 | na | 6737 | -0.279 | 0.2099 | No |
| 100 | CREB5 | na | 6908 | -0.297 | 0.2010 | No |
| 101 | NRF1 | na | 7079 | -0.317 | 0.1924 | No |
| 102 | AP2B1 | na | 7237 | -0.334 | 0.1853 | No |
| 103 | CLTA | na | 7294 | -0.340 | 0.1868 | No |
| 104 | PLCB1 | na | 7317 | -0.343 | 0.1912 | No |
| 105 | DCTN4 | na | 7416 | -0.353 | 0.1894 | No |
| 106 | AP2M1 | na | 7717 | -0.386 | 0.1712 | No |
| 107 | EP300 | na | 7828 | -0.398 | 0.1691 | No |
| 108 | CLTCL1 | na | 7878 | -0.405 | 0.1724 | No |
| 109 | ITPR1 | na | 7928 | -0.411 | 0.1758 | No |
| 110 | POLR2B | na | 8116 | -0.436 | 0.1680 | No |
| 111 | APAF1 | na | 8687 | -0.512 | 0.1293 | No |
| 112 | SIN3A | na | 8777 | -0.522 | 0.1313 | No |
| 113 | CREB3L1 | na | 8939 | -0.540 | 0.1276 | No |
| 114 | DNAH3 | na | 9022 | -0.551 | 0.1308 | No |
| 115 | CLTC | na | 9371 | -0.610 | 0.1126 | No |
| 116 | HTT | na | 9425 | -0.619 | 0.1194 | No |
| 117 | RCOR1 | na | 9725 | -0.664 | 0.1063 | No |
| 118 | VDAC1 | na | 10060 | -0.721 | 0.0914 | No |
| 119 | TAF4 | na | 10163 | -0.743 | 0.0963 | No |
| 120 | PLCB2 | na | 10772 | -0.872 | 0.0610 | No |
| 121 | CREB1 | na | 11301 | -1.038 | 0.0355 | No |
| 122 | CREB3L2 | na | 11794 | -1.322 | 0.0182 | No |
Table: GSEA details [plain text format]

  

Fig 2: KEGG\_HUNTINGTONS\_DISEASE      
 Blue-Pink O' Gram in the Space of the Analyzed GeneSet

  

Fig 3: KEGG\_HUNTINGTONS\_DISEASE: Random ES distribution      
 Gene set null distribution of ES for **KEGG\_HUNTINGTONS\_DISEASE**
